# Surface Modification of Polyamides by Gaseous Plasma—Review and Scientific Challenges

**DOI:** 10.3390/polym12123020

**Published:** 2020-12-17

**Authors:** Gregor Primc

**Affiliations:** Department of Surface Engineering, Jozef Stefan Institute, Jamova Cesta 39, 1000 Ljubljana, Slovenia; gregor.primc@ijs.si

**Keywords:** polyamide, gaseous plasma, water contact angle, XPS

## Abstract

A review of the most significant scientific achievements in the field of surface modification of polyamides by non-equilibrium plasma treatments is presented. Most authors employed atmospheric pressure discharges and reported improved wettability. The super-hydrophilic surface finish was only achieved using a low-pressure plasma reactor and prolonged treatment time, enabling both the nanostructuring and functionalization with polar functional groups. The average increase of the oxygen concentration as probed by XPS was about 10 at%, while the changes in nitrogen concentrations were marginal in almost all cases. The final static water contact angle decreased with the increasing treatment time, and the oxygen concentration decreased with the increasing discharge power. The need for plasma characterization for the interpretation of experimental results is stressed.

## 1. Introduction

Polyamides (PA) are thermoplastics of good thermal stability, high melting point, excellent durability and mechanical properties, and low permeability for oxygen. They are used as engineering plastics in the automotive industry, electrical industry and electronics, packaging, and synthesizing ropes, textiles and membranes. Polyamide is among the most promising materials for vehicle weight reduction by replacing metal parts in the automotive industry due to the ease of mass production and molding. The high demand from the automotive industry is currently the primary driving force of the global polyamide market. Despite its good dyeability, the printability or ability to adhere to other materials is limited due to the relatively poor wettability. Similar to most other polymers, the static contact angle of a water droplet is somehow below 90° for flat surfaces, therefore, the polar coatings do not adhere sufficiently. The surface properties should be altered to ensure better wettability. A typical method for improving the wettability of polymers is the treatment with non-equilibrium gaseous plasma. A product made from or containing polyamide is exposed to reactive gaseous particles and radiation from gaseous plasma. Depending on discharge parameters, the plasma contains various reactive gaseous species, as well as radiation in a broad range from visible to deep ultraviolet (UV) range. Furthermore, the polymer is also subject to bombardment with positively charged ions of kinetic energy from a few eV to several 100 or even 1000 eV, depending on the type of discharge used for sustaining gaseous plasma. The reactive species interact chemically with the surface of the polymer. The bombardment with ions adds to the intensity of chemical reactions, and the UV radiation causes bond scission on the surface and the sub-surface layer. The surface finish obtained upon the plasma treatment is the consequence of all these effects. Different authors reported plasma treatments using various experimental setups, and the results scatter significantly. This paper aims to review recent advances in the plasma treatment of polyamides, drawing correlations between the treatment parameters and surface finish, as well as the identification of future research that should be performed to achieve the desired surface finish in a highly predictable and repeatable manner.

## 2. Literature Survey

The surface modification of polymers is usually studied using smooth and flat samples. Such samples are useful due to the peculiarities of the most common technique for monitoring the evolution of surface functional groups—X-ray photoelectron spectroscopy (XPS). Namely, the XPS spectra are best interpreted if acquired from smooth surfaces. Mandolfino et al. [1] performed systematic research on the plasma activation of PA6 and PA6.6 using a low-pressure gaseous plasma. The plasma reactor was powered with a radiofrequency (RF) supply of adjustable powers up to about 200 W. The treatment was performed with a plasma sustained in air, argon, oxygen, and a mixture of 50% argon and 50% oxygen. Samples of polymer sheets were carefully cleaned with acetone to remove any organic surface contaminants before the plasma treatments. The initial water contact angles (WCAs) of pre-treated samples were 51° and 49° for PA6.6 and PA6, respectively. The plasma exposure time was varied up to 10 min. The evolution of the WCA versus plasma treatment time was studied in detail using air plasma. In the case of PA6 and discharge power of 50 W, the samples assumed a WCA of just about 20° after 1 min of plasma treatment. The prolonged treatment did not have any effect on the water contact angle. When the discharge power was 150 W, the WCA dropped to 32° after 10 s of plasma treatment and remained at about 23° up to 3 min. Then, the WCA started to decrease further until a super-hydrophilic surface finish was observed after 10 min. When the authors used 200 W, no plateau was observed on the curve, but the super-hydrophilic finish occurred even at 1 min of plasma treatment. The super-hydrophilic finish is usually explained by a combined effect of functionalization with polar functional groups and rich morphology on the sub-micrometer scale. The behavior of the WCA for different discharge powers, as reported by Mandolfino et al. [1], is explained by the extensive etching of PA6 at elevated powers. The treatment of smooth polymer surfaces with a low-pressure plasma often causes the formation of periodical structures on the surface, which has been explained by several different mechanisms [2,3,4,5,6,7]. The super-hydrophilic surface finish occurs within relatively narrow limits of experimental conditions [8]. Therefore, it is not surprising that the paper by Mandolfino et al. [1] is the only report on the super-hydrophilic surface finish of PA6.

Similar results were reported by Mandolfino et al. [1] for PA6.6, except that the initial functionalization was a bit more rapid than in the case of PA6. Adhesion of a bi-component acrylic adhesive was studied versus the discharge power and treatment times. Even a brief treatment by gaseous plasma caused a significant increase in the shear strength. Although the WCA for PA6 treated with plasma at 50 W for 10 s remained reasonably intact, the shear strength increased by a factor of 3. Longer treatment times caused even better adhesion of the glue. The best results in terms of shear strength were observed for moderate discharge powers and treatment times; two opposite effects often explain such a maximum in the shear strength versus the treatment parameters. The first one is an increase in surface wettability (functional groups and morphology) with increasing plasma treatment intensity. The second one is a decrease in wettability due to the thermal effects. Namely, the increased discharge power or treatment time (or both) causes heating of the polymer sample and thus rapid loss of the surface functional groups. The heating rate depends on the polymer materials’ roughness and becomes large already at low discharge powers when a polymer is in the form of a powder [9].

Interestingly, the best shear strength at given experimental conditions was always observed for air plasma treatment, although the WCA was relatively moderate compared to other gases. For example, the WCA of PA6 treated with argon plasma at 150 W for 180 s was as low as 3°. At the same treatment time and air as the working gas, the WCA was still 21°.

**Figure 1:**
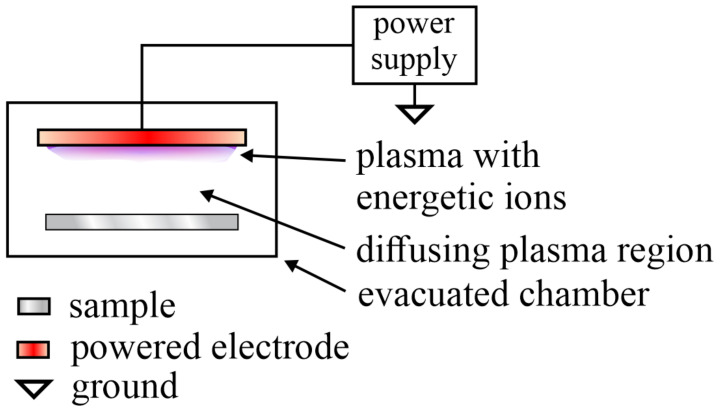
Schematic of the plasma reactor useful for the treatment of polyamide materials with a low-pressure gaseous plasma.

A low-pressure plasma reactor was also used for the pre-treatment of PA6 discs to increase the adhesion properties [10]. The plasma was sustained in argon at the pressure of 40 Pa. The PA6 discs were washed in an ultrasonic bath with ethanol for 10 min, then dried thoroughly at 80 °C for one day. The authors did not reveal the plasma treatment time, but the WCA dropped from 64 to 31°. The XPS showed a small increase in both oxygen and nitrogen concentrations on the surface of the plasma-treated discs, and the peel strength remained practically intact. The plasma treatment, however, was found beneficial for the adhesion of a silane coupling agent, which assured excellent joints between the PA6 and rubber discs. Namely, the peel strength increased from about 6 up to 80 N/cm. The adhesion mechanism and the reactions with the coupling agent are elaborated in [10]. The papers by Mandolfino et al. [1] and Sang et al. [10] are the only scientific documents on the application of low-pressure plasma reactors. Although the configurations vary, both groups applied the plasma reactors shown schematically in Figure 1. The energetic particles are concentrated next to the powered electrode, and the entire volume of the reactor is filled with a rather uniform diffusing plasma. The samples are placed away from the powered electrode to prevent overheating.

Due to the more straightforward configuration, atmospheric pressure plasmas are more popular for treating polyamide foils or textiles than the low-pressure ones. A recent review of such methods for treating packaging materials was prepared by Tyuftin and Kerry [11]. Various research groups have performed detailed studies. Hanusova et al. [12] used two atmospheric pressure plasma sources: A coplanar dielectric barrier discharge (DBD) and a corona discharge with air as the processing gas. The investigated foils were made of PA12. No pre-treatment of the foils was reported by Hanusova et al. [12]. The WCA of an untreated PA12 was 63°. Both plasma treatments caused a significant decrease in the WCA. The difference between the two plasma treatments was relatively marginal, and the WCA of about 30° was obtained already after 5 s of plasma treatment in both cases. XPS was used to detect the evolution of the surface composition. The samples were probed after 5, 10, and 15 s of plasma treatment. The oxygen concentration, as determined by XPS on the surface of virgin PA12, was 28 at%. The DBD discharge enabled an increase in the O concentration to 38 at% for 5 s, 36 at% for 10 s, and 40 at% for 15 s of plasma treatment. Interestingly, as revealed from WCA, the wettability did not follow the evolution of the oxygen concentration. A somehow better correlation between the concentration of oxygen on the surface of the PA12 film and the WCA was observed for the corona treatment. In this case, the oxygen concentration remained practically unchanged for all treatment times, the same as the WCA. The concentration of nitrogen was also probed by XPS. It was found to be about 3 at% for the untreated material and increased to 4 at% for the case of DBD treatment, and 5 at% for the corona discharge treatment. The hydrophobic recovery was also studied in detail both by WCA and XPS. The WCA started increasing soon after the plasma treatment and reached a value of roughly 50° after a few days. Interestingly, the oxygen concentration after 1 day of aging dropped below the value of the untreated material, although the WCA revealed that a moderate hydrophilicity persisted. The oxygen concentration for the PA12 foil treated by the DBD plasma, on the other hand, remained fairly intact for the first few days of aging and persisted at 33 at% even after 14 days.

A similar diffuse coplanar DBD plasma sustained in the air was also used by Károly et al. [13]. The authors used PA6 foil to study adhesion properties using commercial glues. The polymer surface was first polished with a silicon carbide abrasive paper under wet conditions, followed by dry polishing. The final pre-treatment was performed with distilled water and ethanol. The DBD plasma was powered with a 15 kHz AC supply with a peak-to-peak voltage of approximately 20 kV and the plasma treatment time of 30 s was adopted. The adhesion between the plasma-treated polymer and other substrates was measured for the acrylic-based, two-component glue, and epoxy adhesive. The WCA of the pre-treated PA6 was 70°. A half-minute plasma treatment caused a drop to 28°, while a WCA of 21° was observed after 180 s of treatment. The initial oxygen concentration probed by XPS was about 13 at% and increased to 23 at% after 1 min of plasma treatment. The adhesive shear strength depended on the type of glue rather than on the surface finish. For example, the shear strength remained practically unchanged for glue type Loctite 406, but increased by a factor of two for Loctite 330.

A somehow different variety of DBD discharges in the random filamentary mode and sustained in the air was applied by Borcia et al. [14]. The discharge was powered at a frequency of 80 kHz and peak-to-peak voltage of about 15 kV. Several types of polyamide foils were probed. No pre-treatment of the foils was reported by Borcia et al. [14]. The original WCAs were 69, 80, and 102° for PA6, PA6.6, and PA12, respectively. The treatment time was varied between 0.1 and 5 s. Even a 0.1 s plasma treatment caused the WCA to decrease to 34, 48, and 67° for PA6, PA6.6, and PA12, respectively. The prolonged treatment time was beneficial, since the WCA decreased monotonously with the increasing treatment time. As probed by XPS, the concentration of oxygen increased monotonously with the increasing treatment time, but the exact behavior was different from the wettability investigated by WCA. Therefore, the results of Borcia et al. [14] indicate an extremely rapid functionalization of polyamide films using the DBD plasma, which consists of numerous stochastic plasma filaments.

The same authors also employed helium in order to treat polymeric foils with gaseous plasma. Commercial foils of PA6 were pre-treated to remove low molecular weight oligomers by rinsing in propanol for 10 min at room temperature, followed by vacuum drying [15]. In this paper, they used the discharge frequency of 13.5 kHz and peak-to-peak voltage of 28 kV. Helium was introduced into an inner electrode gap, therefore, a plasma plume expanded between the powered and grounded electrode. Despite using pure helium, the authors observed a significant increase in oxygen concentration as probed by XPS. The oxygen concentration of untreated samples was about 14 at% and increased to about 24 at% for both 10 and 30 s treatments. Fascinating, the oxygen concentration decreased to only 20 at% for the treatment time of 1 min. The wettability was expressed in terms of the adhesion work, which was about 100 mJ/m^2^. Almost irrespective of the plasma treatment duration, the adhesion work increased to about 136 mJ/m^2^ for the plasma-treated samples. However, differences were observed in the hydrophobic recovery. Borcia et al. [15] clearly showed that the hydrophobic recovery, in this particular case, was much more pronounced when polyamide foils were functionalized by short plasma treatments.

**Figure 2:**
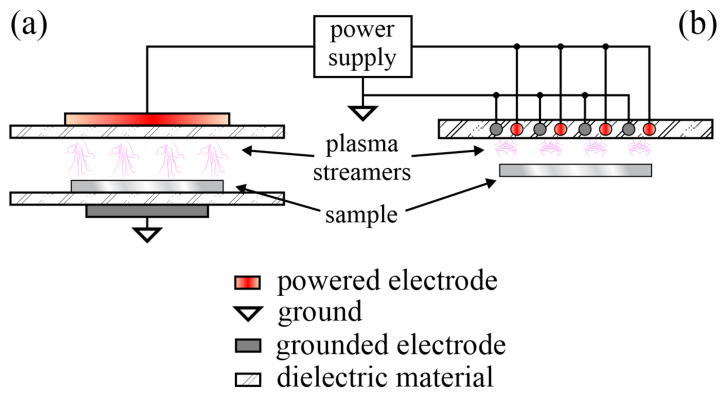
Schematic of atmospheric pressure dielectric barrier discharge in a classical (**a**) and coplanar (**b**) mode.

Either classical or coplanar dielectric barrier discharges (DBD) were used to treat PA materials at an atmospheric pressure. Unlike the low-pressure configuration where a rather uniform plasma occupies a large volume, as shown in Figure 1, the DBD produces temporally and spatially constrained streamers. The schematic of the experimental setups useful for the treatment of PA samples at an atmospheric pressure is shown in Figure 2. Numerous streamers (localized dense plasma of short duration) spread from the electrodes and influence the surface finish of polymer materials. The sample is placed between the electrodes (usually plane-parallel) in the classical mode, whereas in the coplanar mode the sample is above the plasma streamers.

Gao et al. studied the behavior of PA6 films using atmospheric pressure plasma treatments. The specimens were soaked in acetone for 10 min and then dried in a desiccator for 24 h at room temperature to remove finishes and surface contamination. In one paper [16], they used helium discharge with oxygen admixture. The plasma was powered with a radiofrequency generator at the frequency of 13.56 MHz, therefore, the plasma was in a continuous mode. The plasma treatment time was 4 s. Pure helium and helium with 1 or 2% oxygen admixture were used as working gases. The initial WCA of the as-received PA6 foil was 76°, and it decreased slightly for the case of the treatment with pure helium and more intensively for the case of oxygen addition. Moreover, the morphology of the film changed with the increasing addition of oxygen into the gas mixture. The original oxygen and nitrogen concentrations, as probed by XPS, were 15 and 8 at%, respectively. Even the treatment with pure helium plasma caused a significant increase of both oxygen and nitrogen concentrations in the surface film. The nitrogen concentration increased by more than 5 at%, representing one of the highest reported increase among all cited authors. An addition of 1% of oxygen into the gas mixture resulted in a nitrogen concentration of 10 at%, therefore, it increased slightly compared to the untreated samples. In contrast, the further addition of oxygen caused a weaker functionalization with nitrogen, but the concentration remained higher than for the untreated sample. An intriguing behavior was also observed for the oxygen concentration in the surface film. Namely, pure helium plasma caused the O concentration of 26 at%. An admixture of 1% oxygen resulted in 33 at%, while in the case of 2% oxygen admixture to helium plasma, it was 36 at%. The authors also measured the weight loss of the films, which they ascribed to the plasma treatment. Even the pure helium plasma treatment caused a measurable loss of weight, and the loss increased with the increasing oxygen concentration. The peel strength was also measured versus plasma treatment time. In this case, the treatment times were up to 180 s. The original peel strength was 1.7 N/cm, and it increased for all gases, but the best results were observed after the prolonged treatment with helium and 2% oxygen admixture plasma, where the peel strength was the largest at the value of 3.8 N/cm.

Gao [17] also reported the plasma treatment of PA6 films using the same plasma source as in [16] and a plasma treatment time of 30 s. The etching rate, in this case, was expressed in nm/s and the value was about 1.85 for pure helium plasma and 2.65 for a mixture of helium with 1% of oxygen. In this case, the peel strength of an untreated material was 1.8 N/cm. Half a minute treatment with helium plasma resulted in the peel strength of about 2.25 N/cm, while the strength was 2.5 N/cm for the case of helium plasma with 1% oxygen admixture. The reported results on the peel strength in [17] are almost the same as in [16]. The same applies to the chemical composition, as revealed by XPS.

The influence of moisture upon treatment of PA6 films with helium plasma with 1% oxygen at an atmospheric pressure using the same device as in [16,17] was published as in [18]. The etching rate was found as high as 8 nm/s in the case of 9% relative humidity in plasma sustained at the power of 80 W. The same discharge parameters with a relative humidity of 1.6% only resulted in the etching rate of approximately 5 nm/s. The discharge power was the crucial parameter for etching, as the reported rate at the power of 40 W was as low as about 0.5 nm/s, for the case of low relative humidity. The WCAs were also determined after the treatment time of 3 min. The hydrophilicity slightly increased for all treatment parameters. However, the best results with the WCA of 40° were observed for the case of high relative humidity and high discharge power. Similar, if not identical results, were also published in [19].

Moreover, Gao et al. [20] used a mixture of helium and CF_4_ to treat PA6 films using the same experimental setup as in [16,17,18,19]. In this case, the etching rate depended significantly on the plasma treatment time. The reported etching rates are 3.0, 2.6, and 1.9 nm/s for the treatment times of 30, 60, and 90 s, respectively. The WCA dropped from the original 76° to about 27° for half a minute of the plasma treatment, but the prolonged treatment caused an increase of the WCA. At the treatment time of 90 s, the WCA was already about 100°. As the treatment time increased, the surface morphology developed as well. The average roughness, as deduced from AFM imaging, was 12 nm for the untreated material and as large as 36 nm for the material treated in the He/CF_4_ plasma for 90 s. Fluorine was observed by XPS on the surface of the plasma-treated samples. The fluorine concentration was marginal for the treatment time of 30 s, but increased to approximately 10 at% for the treatment time of 90 s. Noteworthy, both oxygen and nitrogen concentrations, as revealed from the XPS survey spectra, were larger for plasma-treated samples than for the untreated sample. An interesting behavior was also reported for the peel strength.

Pappas et al. [21] treated both PA6 films and fibers using an atmospheric pressure glow discharge operating at the frequency of 90 kHz. Helium was used to ignite the discharge and nitrogen, or acetylene was added further on. The operating power was 850 W. The WCA on an untreated material was 76°, and it decreased to 58° upon treatment with the N_2_/He plasma for 10 s. A rather interesting behavior of the WCA versus the treatment time is revealed in this paper and aging was also tackled. The slowest hydrophobic recovery was observed for PA6 foils treated for the most extended treatment time. The WCA increased to 55° after 1 month of aging at ambient conditions for the case of the 10 s plasma treatment. The same material treated by the plasma for 0.6 s only assumed the WCA of 65° after 1 month of aging. The WCA was still lower than for the original samples.

Polyamide 12 films were also treated by gaseous plasma sustained at an atmospheric pressure using a microwave (MW) plasma source [22]. The dense plasma was sustained within a discharge cavity using surface waves, and a plasma plume also expanded outside the cavity due to the continuous supply of argon. Different admixtures of oxygen and nitrogen were used to facilitate chemical reactions between the plasma jet and polymer samples. The treatment time was between 25 ms and 1 s. Although no pre-treatment was reported by Hnilica et al. [22], the WCA of untreated samples was about 80°. Even the shortest treatment time caused a decrease of the WCA to about 28°, while a further treatment resulted in a slightly lower contact angle. For example, at 1 s, the WCA was about 22°. Hnilica also provided lateral profiles and the diameter of the affected area was about 1 cm. The interface between the affected and original surface wettability was rather sharp. The oxygen concentration, as probed by XPS, marginally increased after 25 ms of plasma treatment. However, 1 s of plasma treatment caused the oxygen concentration to increase to 31 at% for a sample treated with argon, 34 at% for the treatment with argon mixed with 2% oxygen, and 36 at% for the treatment with argon mixed with 2% nitrogen. The concentration of nitrogen did not change significantly depending on the type of gas or the treatment time. The results presented by Hnilica, therefore, reveal extremely fast hydrophilization of PA12. The treatment times well below a second are very useful for industrial-scale modification of surface wettability, especially when the treatment is performed continuously.

The surface properties of polyamides can also be tailored using energetic ions. A useful device was applied by Kalácska et al. [23]. Samples of extruded PA6 of disc shape were polished with a silicon carbide abrasive paper and subsequently cleaned in an ultrasonic bath in distilled water and ethanol. The final stage of pre-treatment was drying in pure nitrogen at an atmospheric pressure. Samples were immersed into a high vacuum chamber, where the plasma was generated by a 27.13 MHz RF generator in relatively pure nitrogen. The estimated discharge power was 150 W, and the working pressure was 0.4 Pa. Samples were placed on the additionally biased electrode with the voltage of −30 kV and treated at conditions where the achieved fluence was 3 × 10^13^ ions/m^2^. The oxygen concentration in the surface film increased by 24 at%, while the nitrogen content increased by 13 at% upon the nitrogen ion treatment. The WCA decreased from the original 60° down to 36°. The friction coefficient of the treated samples was fairly low at the beginning. Under dry sliding, the increased surface energy caused increased adhesion, associated with forming a transfer layer on the steel disc. A decreased friction was observed upon the run-out type lubrication test, explained by the increased oil retention on the treated surface.

PA66 foils were also treated by the atmospheric pressure DBD air plasma by Kuzminova et al. [24] using a semi-continuous mode. The power supply operated at the frequency of 22.5 kHz and discharge power of 30 W. The configuration was very similar to that adopted by Karoly et al. [13]. The treatment time ranged from 0.5 s to 0.5 min. The roughness, as determined by AFM, remained quite unchanged for the treatment time up to 15 s and increased significantly with the prolonged treatment times. The surface composition, as probed by XPS, was reasonably close to theoretical (C:O:N = 70:15:15) for untreated samples. Even 0.5 s of plasma treatment caused an increase of the oxygen concentration to 22 at%. The oxygen concentration increased rather monotonically with the increasing treatment time and reached 33 at% after half-a-minute of plasma treatment. The nitrogen concentration also increased with the increasing treatment time but only for a couple of at%. High-resolution XPS C1s peaks revealed a gradual increase of the carboxyl groups on the PA66 samples. The WCA dropped from the original 64° to about 30° even for the first 0.5 s treatment. With the increasing treatment time, it slowly decreased until about 25° was observed after half a minute of plasma treatment. The dispersive component of the surface energy remained practically unchanged during the plasma treatment. However, the polar component jumped to about 27 mJ/m^2^ even for the shortest treatment time, indicating swift surface activation upon treatment by the DBD plasma. Hydrophobic recovery was marginal during the first 100 h. Optical, thermal, and mechanical properties remained practically intact, irrespective of the plasma treatment time.

Plasma treatments were also found useful for the modification of polyamide fibers, textiles, or membranes. The improved dyeing ability of PA66 fabrics was reported by Oliveira et al. [25]. Fabrics with a warp density of 42 threads/cm, weft density of 30 threads/cm, and mass per surface area of 95 g/m^2^ were pre-washed with a 1 g/L of non-ionic detergent solution at 30 °C for 30 min and then rinsed in water for another 15 min. A commercial DBD plasma reactor was powered with a 10 kV source operating at 40 kHz. The fabric was treated in a continuous mode at the velocity of 4 m/min using 1 kW of discharge power. The corresponding treatment time was about 0.5 s. Such a short treatment time caused a significant modification of the surface composition. XPS spectra revealed that the oxygen concentration increased from 16 to 28 at% and the nitrogen concentration from 9 to 11 at%. The untreated fabric exhibited hydrophobic properties with WCA as large as 133°. The plasma treatment resulted in a WCA of 21° and moderate hydrophobic recovery upon aging. The WCA increased to about 23° after 1 day of aging at ambient conditions, 42° after 1 week, and 80° after a month. The plasma treatment caused a significant increase in the polar component of surface energy, from 1 to 73 mJ/m^2^. In comparison, the dispersive component, as calculated using the Wu method [26,27], decreased from 10 to 7 mJ/m^2^. The ability of dyeing was improved significantly, and the fastness properties persisted even after prolonged washing. Oliveira et al. also disclosed a comprehensive dyeing mechanism [25]. After the plasma treatment, the PA66 displayed a significant amount of oxygen covalently bonded to the surface. The presence of microchannels promoted the diffusion of the dye into the fibers.

In another paper [28], Oliveira et al. described the influence of DBD plasma on the trichromic dyeing process of PA66 fabric and the reuse of the generated effluents for new dyeing processes. Fabric with a warp density of 40 threads/cm, weft density of 30 threads/cm, and mass per surface area of 95 g/m^2^ was pre-washed and treated by DBD as in [25]. The static WCA of the untreated material was as high as 146°. The plasma treatment at a moderate discharge power density caused a rapid decrease of the static WCA until the power density of 2500 Wmin/m^2^, at which it became immeasurable, since the plasma-activated fabric absorbed the water droplet before the measurement could be performed. In contrast to [25], the XPS results indicated only moderate functionalization of the fabric’s surface as the oxygen content increased from 18 to 20 at% and nitrogen from 8 to 10 at%. The chemical composition was also probed by energy-dispersed X-ray spectroscopy (EDS). The method revealed an increase in the oxygen content from 23 to 25 at% and in nitrogen from 10 to 11 at%. The excellent agreement between XPS and EDS results was explained by the substantial incorporation of O and N atoms onto the fabric’s surface. The difference in nitrogen content was explained by a surface cleaning effect that allowed the bulk material’s fingerprint to appear on the spectra.

In yet another paper, Oliveira et al. focused on the whiteness degree and the tensile strength of polyamide fabric of various warp and weft densities [29]. The static WCA of untreated material was as high as 153° for the PA66 fabric of warp density of 8 threads/cm, weft density of 18 threads/cm, and mass per surface area of 95 g/m^2^. The static WCA dropped to 75° after the treatment by the DBD plasma of 500 Wmin/m^2^ power density. The plasma treatment with a power density of 1500 Wmin/m^2^ caused a static WCA of 53°, while higher power densities caused an immediate absorption of a water droplet, therefore, no static WCA could be measured. The water absorption time was estimated to about 1 s for the well-activated PA66 samples. Both XPS and EDS were used to monitor the elemental composition. The XPS showed a significant increase in the oxygen content after treating different fabrics with a plasma sustained at the power density of 2500 Wmin/m^2^—from 17 at% (for the untreated fabric) to about 28 at%. EDS showed a much smaller increase, i.e., from about 23 to 25 at%. A more considerable probing depth of EDS could explain such a discrepancy. The tensile strength and elongation remained unchanged after the plasma treatments.

A DBD plasma was also used for surface modification of PA66 by Bessada et al. [30]. A commercially available PA66 woven fabric of mass per surface area of 240 g/m^2^ was treated in a continuous mode without any pre-treatment. The DBD was powered with a 40 kHz supply of peak voltage ~10 kV. The discharge power was varied between 500 and 1500 W, and the speed of fabric passed the DBD between 5 and 20 m/min. The DBD was operating in the filamentary mode, the same as many other DBDs operating at this frequency. Unlike Oliveira [25,28,29], Bessada et al. [30] found only modest wettability changes. The original static WCA was just below 80° and dropped to any value between 50 and 75°, depending on the treatment parameters. The slowest speed of fabric through the DBD caused the best wettability, which is explained by the prolonged plasma exposure time. Interestingly, no statistically significant difference was observed between discharge powers of 1000 and 1500 W. Three consecutive passages of fabric under the discharge caused no significant improvement of the surface wettability. The water droplets were absorbed fast after the plasma treatment—the WCA became immeasurable about 2 s after the droplet’s deposition onto the activated fabric. A moderate hydrophobic recovery was also reported along with a marginal increase of the O and N concentrations as deduced from XPS. From this perspective, the results are similar to those reported by Oliveira [28], except for the absolute values.

Li et al. [31] also used an atmospheric-pressure DBD for treatment of the PA66 fabric with air plasma. However, they used a pulsed DC source to power the discharge, and the discharge chamber was cylindrical. The pulse rise-time was 30 ns only, and the peak voltage as high as 70 kV. The duration of plasma during each pulse was estimated at approximately 750 ns. The repetition time was variable between about 2 ms and 1 s. Samples were first sterilized by alcohol, then cleaned with deionized water and dried before the plasma treatment. After a few seconds of plasma treatment, originally smooth fibers assumed rich morphology, and after 1 min of plasma treatment, a well-defined cone-like morphology appeared. XPS was used to study the surface composition. The untreated samples contained 14 at% O and 8 at% N. A 10-s plasma treatment caused an increase in oxygen concentration to about 16 at%, but nitrogen concentration dropped for about 1 at%. A 1-min plasma treatment resulted in 18 at% O and 9 at% N in the surface film of PA66 as probed by XPS. The same group published the same results on the surface characterization of PA66 in [32] and added a detailed study on the disinfection efficiency using *Escherichia coli* HT115 as model microorganisms.

All the above-cited authors used high impedance discharges for the plasma treatment of polyamide fabric at an atmospheric pressure. Pavlinak et al. [33], however, employed a microwave (MW) discharge, similar to that reported by Hnilica et al. [22]. PA6 nanofibers were synthesized by electrospinning and exposed to an MW plasma jet sustained in pure argon or a mixture of argon and 2 vol% oxygen at an atmospheric pressure. As expected, the nanofibers melted upon exposure to MW plasma sustained in the continuous mode. The samples passed the vicinity of the MW plasma jet at the speed of 12 mm/s. The surface composition, as determined from the XPS survey spectra, was studied versus the number of passes. The original O and N concentrations in the surface film were 11 at%. The concentrations changed after passing the vicinity of the MW plasma jet. One pass caused an increase in oxygen concentration in the surface film to 18 and 15 at% for the cases of pure Ar and Ar + 2 vol%O_2_, respectively. Simultaneously, the N concentration decreased to 6 and 9 at%, respectively. The unanticipated result may be explained by the presence of water vapor in the effluent regime of the MW plasma jet. Pavlinak et al. [33] also showed that the O-concentration increased by increasing the number of plasma-passes. After about 10 passes, the O-concentration stabilized at about 22 at% for both Ar and Ar + 2 vol%O_2_ treatments. Fibers have melted after such treatments. Some samples were also heated for 1 min to 300 °C, without being exposed to the plasma, to check whether the oxidation would occur merely due to thermal oxidation. Although the PA6 nanofibers, synthesized by electrospinning, melted at 300 °C, practically no oxidation was detected by XPS. Therefore, the extensive functionalization observed after the plasma treatment could not be attributed to the high sample temperature. Interestingly, the high concentration of functional groups remained on the surface even though the samples melted upon the plasma treatment. Namely, polar functional groups are usually unstable at high temperatures and decay spontaneously upon heating [34].

Polyamide is also an exciting material for the synthesis of membranes. Boulares-Pender et al. [35] treated nylon membranes of thickness of 0.15 mm and nominal pore sizes of 0.2 µm in a low-pressure plasma reactor using argon, nitrogen, and oxygen gases. The gas pressure was set to about 25 Pa and the flow rate to 20 sccm. The ultimate pressure was about 15 Pa, but the authors did not report the residual atmosphere’s composition. The plasma treatment always caused an increased oxygen concentration as probed by XPS, almost irrespective of the gas or gas mixtures used. The original O-concentration was 12 at%, and it increased to about 18 at% after the plasma treatment. The nitrogen concentration remained somewhat intact at about 11 at%. No carboxyl or similar highly polar functional group was deduced from the high/resolution XPS C1s spectra. The peak attributed to the amide group remained intact irrespective of the type of gas or gas mixture used upon the plasma treatment. Plasma-treated membranes were further treated by electron beams using a home-made electron accelerator operating at about 170 kV. The adsorption of proteins on plasma-treated membranes was also studied by Boulares-Pender et al. [35]. The adsorption of albumin (BSA) was hardly affected by any plasma treatment. However, myoglobin adhered well on all plasma treated membranes, especially when the plasma was sustained in nitrogen or a nitrogen-argon gas mixture. The adsorbed quantity was doubled as compared to the pristine nylon membrane. Lysozyme, on the other hand, completely adhered to the membrane treated by the plasma containing oxygen. The authors found no correlation between the surface functionalities as probed by XPS and adhesion of blood proteins, which is slightly unexpected, considering the earlier works [36].

## 3. Correlations between Surface Finish and Treatment Parameters

The most relevant results reported by different authors are summarized in Table 1. Only the surface finish reported by a good number of authors is included in the summary. An interesting feature revealed by examining Table 1 is a large scattering of the original surface wettability. The WCA of the untreated samples spans from 49 to 153°. In general, the WCA does not depend only on the type of material but also its roughness. From the latter point of view, the WCA should be the highest for untreated samples of rich morphology on the sub-micrometer scale. Unusually, the water contact angles for the same material and morphology scatter significantly, as well. A feasible explanation is the purity of the material, both in terms of intentionally added blends and surface impurities. Unfortunately, not all authors reported details on the pre-cleaning of their samples. The surface impurities indeed influence the initial surface composition and thus the surface energy, but should not affect the final wettability after the plasma treatment, since they are likely to be removed upon the plasma treatment. Many authors did not report the evolution of surface morphology (usually studied by AFM or SEM). Therefore, it is impossible to draw a correlation between the roughness on the sub-micrometer scale and surface wettability.

**Figure 3:**
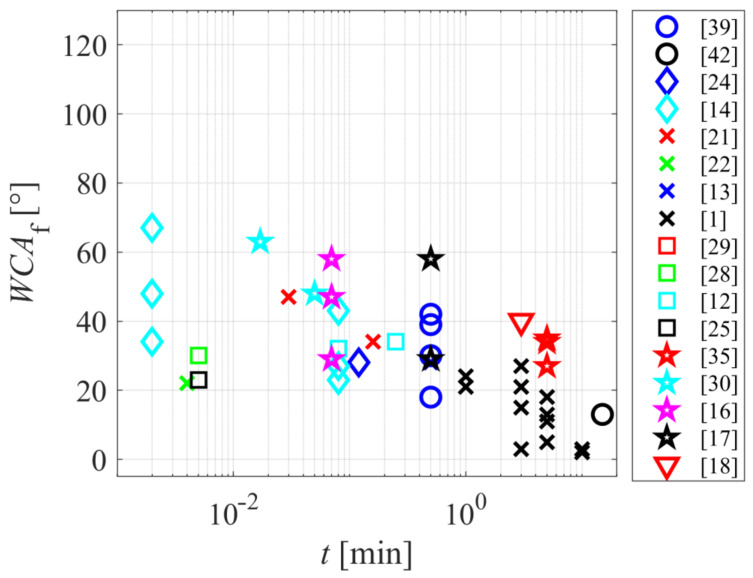
The final water contact angle versus the treatment time.

The final WCA reported by the majority of authors is between 20 and 40°. Figure 3 reveals the final WCA versus the treatment time. Despite the large scattering of the reported results, there is an evident trend—a longer treatment time results in a lower WCA and thus better wettability. The super-hydrophilic effect (WCA below a few degrees) was reported only for treatment times of several minutes. This observation is explained due to the fact that the WCA on a smooth surface cannot be extremely low, but a nanostructured surface finish is needed for the super-hydrophilic effect [8]. Nanostructuring of polymers is a result of non-homogeneous etching. The etching at a reasonable temperature (preferably close to the room temperature) is a slow process since the etching rate is often the order of nanometers per second [45,46]. The combination of rich surface morphology and high concentration of polar functional groups is usually observed only for polymers treated by low-pressure plasma [46,47]. Surprisingly, most authors concentrated their research on atmospheric-pressure plasmas, although they rarely enable a super-hydrophilic surface finish.

**Figure 4:**
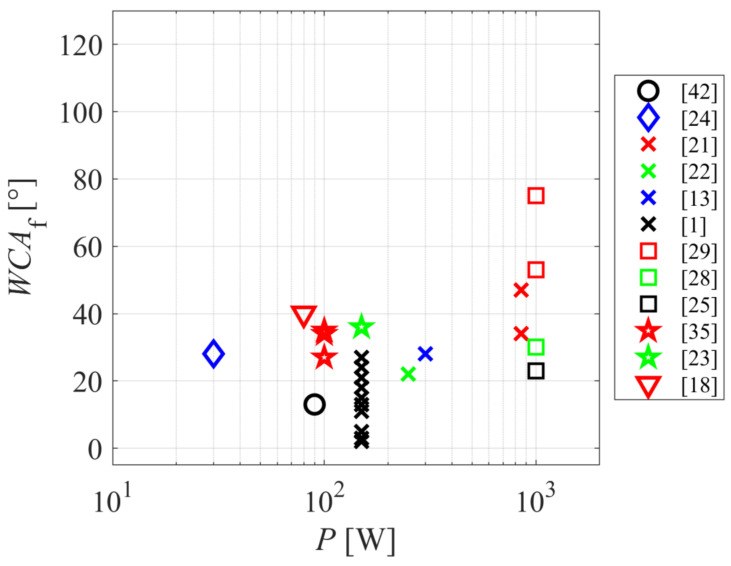
The final water contact angle versus the discharge power.

The relationship between the surface wettability and discharge power is shown in Figure 4. It seems that high discharge powers do not lead to highly wettable surfaces, but moderate powers around 100 W perform better. It should be stressed that the absolute value of the discharge power may not be the best parameter, since the processing parameters depend on numerous other parameters such as the size of the treatment device, the pressure, and many other peculiarities. A more appropriate parameter would be the power applied to the plasma and normalized to the surface area (in the case of one or two-dimensional discharges) or to the plasma volume (in the case of continuous discharges that create the homogeneous plasma in a particular volume).

**Figure 5:**
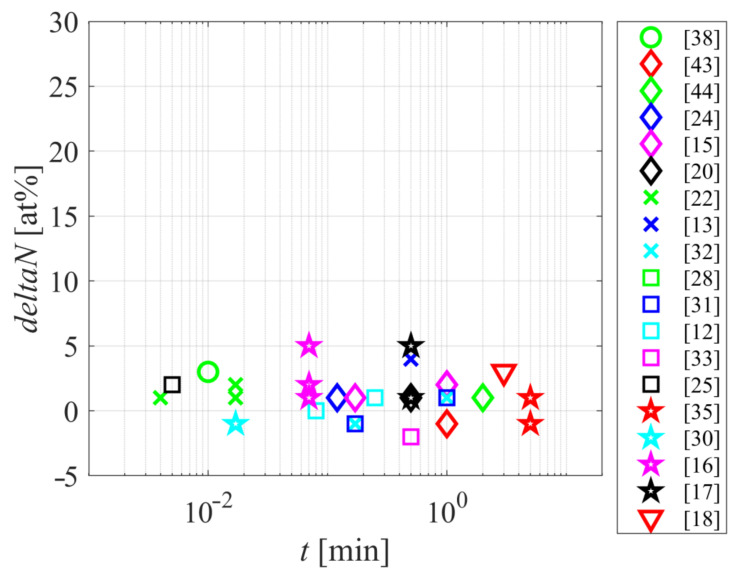
The difference in nitrogen concentration as probed by XPS versus the plasma treatment time.

The majority of authors used XPS for monitoring the modification of surface chemistry. While the XPS survey spectra are acquired in a reasonable time, the acquisition and interpretation of high-resolution spectra may take time, therefore, most authors limited such investigations. Still, most authors represented the concentration of nitrogen, oxygen, and carbon versus the treatment parameters. Figure 5 represents the difference in N-concentration, as probed by XPS, versus the treatment time. Evidently, there is no trend, and the difference is rather small. Some authors reported enrichment in the N-concentration, but many a depletion despite using plasma sustained in the air or even “pure” nitrogen. The functionalization of polyamides with nitrogen functional groups (such as amino groups), therefore, remains a challenge. A feasible explanation for the lack of additional nitrogen on the PA surface is the preferential functionalization with oxygen. It is difficult to assure oxygen-free plasma due to the water vapor, which is likely to be present in low-pressure and atmospheric-pressure plasma reactors. Here, it should be stressed that even a 1 min concentration of water vapor in processing gas will cause the formation of OH radicals upon plasma conditions and the interaction of these radicals with the polymer surface. The effect of such water-vapor on the surface finish of a polymer upon treatment with “pure” argon was recently elaborated in [48].

**Figure 6:**
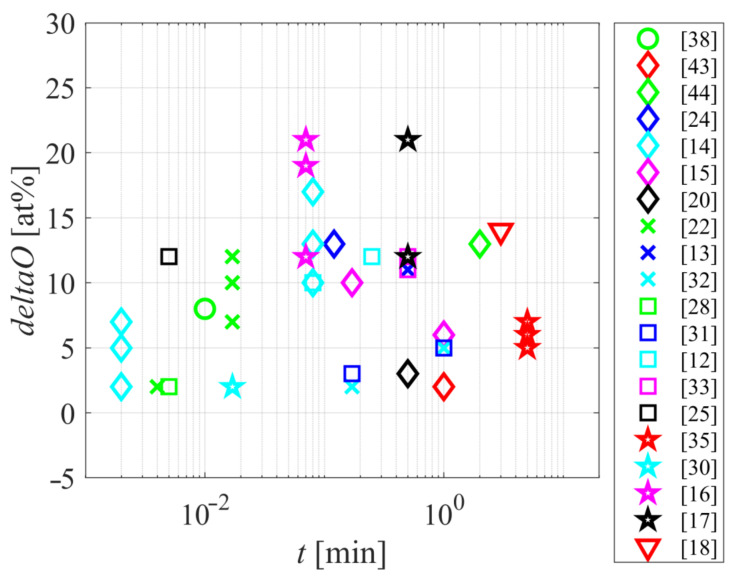
The difference in oxygen concentration as probed by XPS versus the treatment time.

The functionalization with oxygen functional groups is a common consequence of plasma treatment of polyamides. Figure 6 represents the increase of the oxygen content, as probed by XPS, versus the treatment time. Please note the logarithmic scale on the *x*-axis. Figure 6 reveals that all authors reported at least an increase of 2 at%. The average increase is about 10 at%, and it seems that the treatment time is not the factor dictating the concentration of oxygen on the surface of a polyamide sample. Such a rather unexpected fact is explained by the etching of polymer samples whose surfaces have been saturated with oxygen functional groups. The final concentration of oxygen is, therefore, a compromise of surface functionalization by the chemical interaction of reactive species such as O, OH, O_3_, and positively charged ions, and the removal of surface functionalities by releasing CO, CO_2_, and more complex molecules. The etching rate increases with increasing discharge power.

**Figure 7:**
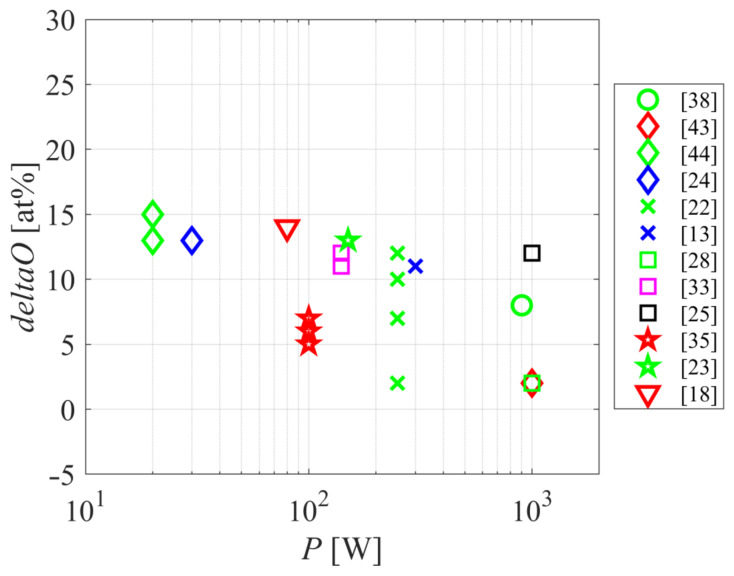
The difference in oxygen concentration as probed by XPS versus the discharge power.

Figure 7 shows the relationship between the oxygen concentration, as probed by XPS and the reported discharge power. The results are scattered due to the experimental setups’ peculiarities, but a general trend is decreasing the O-concentration with the increasing discharge power. A feasible explanation is the extensive etching at elevated powers. The best functionalization should be obtained upon saturation with polar functional groups, as revealed recently, for the case of polystyrene [49]. Such studies have, however, not been performed for polyamides and represent a great scientific challenge.

**Figure 8:**
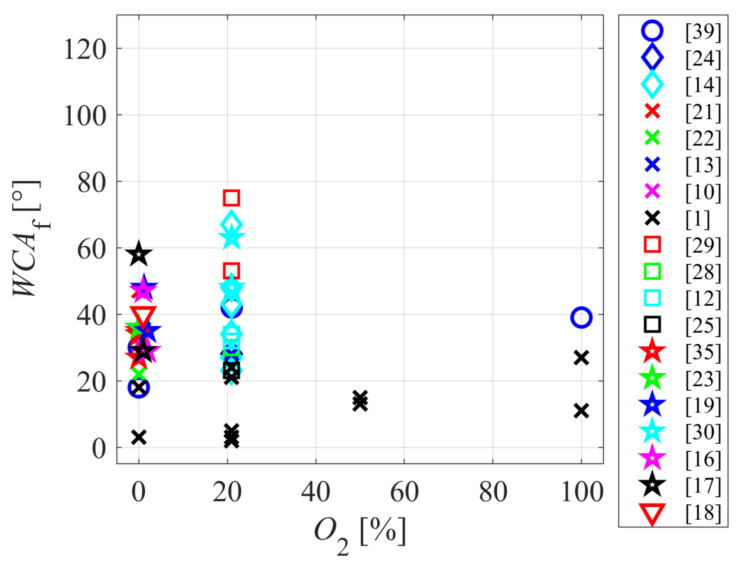
The final water contact angle versus the concentration of oxygen in the original gas mixture.

The concentration of oxygen in the original gas mixture does not seem to be a decisive factor for surface wettability. Figure 8 represents the final water contact angle versus oxygen concentration. Most authors used air as the source gas or a noble gas with a small oxygen admixture. Only a couple of authors employed almost pure oxygen for the plasma treatment of polyamides. The results summarized in Figure 8 show no correlation between the wettability and oxygen concentration in the gas mixture. Therefore, one can conclude that even a small admixture of oxygen in the gas mixture assures a highly wettable surface. This effect is explained by the preferential dissociation of oxygen and/or water vapor in the non-equilibrium gaseous plasma. The group of A. Ricard [50] showed that oxygen atoms extensively dissociate upon plasma conditions due to the rather low dissociation energy (as compared to the nitrogen dissociation energy or excitation energy of Ar or He metastables) and the existence of a couple of long-living molecular metastable states (the “a” and “b” states of neutral O_2_ molecule). The long lifetime of such metastables enables a step-wise dissociation, therefore, the electron threshold energy for a dissociative collision is minimal. As a result, the density of O-atoms or OH radicals in a plasma sustained in other gases is large enough to cause the rapid functionalization of fabrics with oxygen functional groups [51]. Here, it is worth mentioning that the required fluence of oxygen atoms onto a smooth polymer surface for saturation with polar O-rich functional groups is close to 10^21^ m^−2^ [49]. Therefore, the surface is saturated in 1 s of the plasma treatment providing that the O-atom density in the vicinity of the polymer sample being treated is about 10^19^ m^−3^, which is a typical value in many industrial plasma reactors useful for surface activation of polymers [52].

## 4. Conclusions and Roadmap

The survey of recent reports on the plasma functionalization of polyamides for the improved wettability indicates that the surface layer, as probed by XPS, is always enriched in oxygen functional groups irrespective of the type of gas used for plasma treatments. Useful results can be obtained at the treatment time as low as 1 s, providing that the discharge parameters are chosen carefully. Most authors, however, adopted longer treatment times lasting about 1 min. Such treatments cause an increase in oxygen concentration. However, the concentration of nitrogen in the surface film, as probed by XPS, remained relatively intact even when “pure” nitrogen or mixtures of nitrogen with other gases were used to sustain a non-equilibrium gaseous plasma. The concentration of oxygen in the surface film was found inversely proportional to the discharge power, which can be explained by the increased etching of the polymers upon powerful plasma conditions. Thermal effects may play a role in the final concentration of oxygen functional groups. However, only a few authors reported the increased surface temperature due to the exothermic surface reactions such as neutralization of charged particles, bombardment with positively charged ions, the heterogeneous surface association of radicals to stable molecules, and relaxation of any metastables that might appear in the plasma at moderate densities.

Only a handful of authors reported plasma parameters in papers disclosing the plasma activation of polyamides. In an analogy with other polymers, the surface finish should depend on the fluence of reactive gaseous species and ultraviolet radiation. Low fluences should cause functionalization, while higher fluences should cause etching of the already functionalized polymer surface and thus loss of some surface functionalities. Therefore, the scientifically spotless description of the surface phenomena upon the plasma treatment of polyamides should indicate the surface finish versus the fluences of reactive species. Numerous plasma characterization methods are available, and the authors are encouraged to report at least the most crucial parameters, such as densities of highly reactive gaseous species that cause surface modification. Considering the results reviewed in this paper, the density of reactive oxygen species (O-atoms and OH radicals in particular) should be the critical parameter governing the surface finish.

Another scientific challenge is the functionalization of polyamide surfaces with functional groups other than oxygen. The literature survey clearly shows that even 1 min concentrations of oxygen or water vapor in the processing gas caused oxidation of the PA surfaces. The functionalization with other functional groups, therefore, requires an oxygen-free atmosphere, which can be achieved by the careful construction and preparation of experimental setups. Low-pressure plasma reactors should be hermetically tight and bakeable to elevated temperatures (to remove the water adsorbed on surfaces) and pumped with powerful high-vacuum pumps. The atmospheric pressure reactors should also be bakeable, and the diffusion of the surrounding atmosphere into the gaseous discharge should be prevented.

## Figures and Tables

**Table 1:** Summary of the reported results.

Ref.	Gas	*P* [W]	*p* [mbar]	*f* [MHz]	*t* [min]	*O_2_* [%]	Δ*O* [at%]	*N* [at%]	*WCA*_0_ [°]	*WCA*_f_ [°]	Peculiarity	Polymer Type
[37]	O_2_	40	0.5	N/A	0.2	N/A	N/A	N/A	N/A	N/A	N/A	PA
[38]	Air	900	1000	N/A	0.01	21	8	3	N/A	N/A	textile	N/A
[39]	Ar	N/A	1	N/A	0.5	0	N/A	N/A	80	18	tube	N/A
[39]	O_2_	N/A	1	N/A	0.5	100	N/A	N/A	80	39	tube	N/A
[39]	N_2_	N/A	1	N/A	0.5	0	N/A	N/A	80	30	tube	N/A
[39]	Air	N/A	1	N/A	0.5	21	N/A	N/A	80	42	tube	N/A
[40]	Air	900	1000	0.02	0.005	21	N/A	N/A	N/A	N/A	crystal	PA6
[41]	Ar	50	0.2	13.56	N/A	N/A	N/A	N/A	N/A	N/A	N/A	N/A
[42]	N_2_	90	0.015	0.04	15	N/A	N/A	N/A	N/A	13	membrane	N/A
[43]	Air	1000	1000	0.04	1	21	2	−1	N/A	N/A	N/A	PA66
[44]	O_2_	20	0.2	13.56	2	100	13	1	N/A	N/A	N/A	PA12
[44]	O_2_	20	0.2	13.56	30	100	15	0	N/A	N/A	N/A	PA12
[24]	Air	30	1000	0.022	0.12	21	13	1	64	28	foil	PA66
[14]	Air	N/A	1000	0.08	0.002	21	5	N/A	69	34	foil	PA6
[14]	Air	N/A	1000	0.08	0.08	21	10	N/A	69	27	foil	PA6
[14]	Air	N/A	1000	0.08	0.002	21	7	N/A	81	48	foil	PA66
[14]	Air	N/A	1000	0.08	0.08	21	13	N/A	81	23	foil	PA66
[14]	Air	N/A	1000	0.08	0.002	21	2	N/A	102	67	foil	PA12
[14]	Air	N/A	1000	0.08	0.08	21	17	N/A	102	43	foil	PA12
[15]	He	N/A	1000	0.013	0.17	0	10	1	N/A	N/A	foil	PA6
[15]	He	N/A	1000	0.013	1	0	6	2	N/A	N/A	foil	PA6
[20]	He + 1% CF_4_	N/A	1000	13.56	0.5	0	3	1	N/A	N/A	foil	N/A
[21]	N_2_He	850	1000	0.09	0.16	0	N/A	N/A	76	34	foil	PA6
[21]	N_2_He	850	1000	0.09	0.03	0	N/A	N/A	76	47	foil	PA6
[22]	Ar	250	1000	2450	0.004	0	2	1	80	22	foil	PA12
[22]	Ar	250	1000	2450	0.017	0	7	1	80	N/A	foil	PA12
[22]	Ar	250	1000	2450	0.017	2	10	1	80	N/A	foil	PA12
[22]	Ar + 2% N_2_	250	1000	2450	0.017	0	12	2	80	N/A	foil	PA12
[13]	Air	300	1000	0.015	0.5	21	11	4	70	28	foil	PA6
[32]	Air	N/A	1000	N/A	0.17	21	2	−1	N/A	N/A	textile	PA66
[32]	Air	N/A	1000	N/A	1	21	5	1	N/A	N/A	textile	PA66
[10]	Ar	N/A	0.4	N/A	N/A	0	4	−1	64	31	foil	PA6
[1]	Air	150	0.6	13.56	1	21	N/A	N/A	49	24	foil	PA6
[1]	Air	150	0.6	13.56	10	21	N/A	N/A	49	2	foil	PA6
[1]	Air	150	0.6	13.56	1	21	N/A	N/A	51	21	foil	PA66
[1]	Air	150	0.6	13.56	10	21	N/A	N/A	51	3	foil	PA66
[1]	Air	150	0.6	13.56	3	21	N/A	N/A	49	21	foil	PA6
[1]	Ar	150	0.6	13.56	3	0	N/A	N/A	49	3	foil	PA6
[1]	O_2_	150	0.6	13.56	3	100	N/A	N/A	49	27	foil	PA6
[1]	ArO_2_	150	0.6	13.56	3	50	N/A	N/A	49	15	foil	PA6
[1]	Air	150	0.6	13.56	5	21	N/A	N/A	49	5	foil	PA66
[1]	Ar	150	0.6	13.56	5	0	N/A	N/A	49	18	foil	PA66
[1]	O_2_	150	0.6	13.56	5	100	N/A	N/A	49	11	foil	PA66
[1]	ArO_2_	150	0.6	13.56	5	50	N/A	N/A	49	13	foil	PA66
[29]	Air	1000	1000	0.04	0.0002	21	N/A	N/A	153	75	textile	PA66
[29]	Air	1000	1000	0.04	0.0006	21	N/A	N/A	153	53	textile	PA66
[28]	Air	1000	1000	0.04	0.005	21	2	2	146	30	textile	PA66
[31]	Air	N/A	1000	N/A	0.17	21	3	−1	N/A	N/A	textile	PA66
[31]	Air	N/A	1000	N/A	1	21	5	1	N/A	N/A	textile	PA66
[12]	Air	N/A	1000	N/A	0.08	21	10	0	63	32	foil	PA12
[12]	Air	N/A	1000	N/A	0.25	21	12	1	63	34	foil	PA12
[33]	Ar	140	1000	2450	0.5	0	11	−2	N/A	N/A	textile	PA6
[33]	Ar	140	1000	2450	0.5	2	12	−2	N/A	N/A	textile	PA6
[25]	Air	1000	1000	0.04	0.005	21	12	2	133	23	textile	PA66
[35]	N_2_	100	0.1	13.56	5	0	5	1	56	34	membrane	N/A
[35]	Ar	100	0.1	13.56	5	0	6	−1	56	27	membrane	N/A
[35]	O_2_	100	0.1	13.56	5	0	7	1	56	35	membrane	N/A
[23]	N_2_	150	0.004	27.13	N/A	0	13	6	60	36	foil	PA6
[19]	HeH_2_O	N/A	1000	13.56	N/A	1.2	11	1	76	48	foil	PA6
[19]	HeH_2_O	N/A	1000	13.56	N/A	2	23	1	76	35	foil	PA6
[30]	Air	N/A	1000	N/A	0.017	21	2	−1	79	63	textile	PA66
[30]	Air	N/A	1000	N/A	0.05	21	N/A	N/A	79	48	textile	PA66
[16]	He	N/A	1000	13.56	0.07	0	12	5	76	58	foil	PA6
[16]	He	N/A	1000	13.56	0.07	1	19	2	76	47	foil	PA6
[16]	He	N/A	1000	13.56	0.07	2	21	1	76	29	foil	PA6
[17]	He	N/A	1000	13.56	0.5	0	12	5	76	58	foil	PA6
[17]	He	N/A	1000	13.56	0.5	1	21	1	76	29	foil	PA6
[18]	HeH_2_O	80	1000	13.56	3	1	14	3	76	40	foil	PA6
